# Central vein sign and paramagnetic rim lesions in pediatric-onset multiple sclerosis: a systematic review and meta-analysis

**DOI:** 10.1007/s00415-026-14084-6

**Published:** 2026-08-22

**Authors:** Anna de Mauro, Rosa Cortese, Giulia Fadda, Nicola De Stefano, Ludwig Kappos, Maria Pia Sormani, Brenda Banwell, Cristina Granziera, Alessandro Cagol

**Affiliations:** 1 Translational Imaging in Neurology (ThINk) Basel, Department of Biomedical Engineering, Faculty of Medicine, University Hospital Basel and University of Basel, Basel, Switzerland; 2 Department of Medicine, Surgery and Neuroscience, University of Siena, Siena, Italy; 3 Multiple Sclerosis Centre, Departments of Neurology, Clinical Research and Biomedicine, University Hospital and University of Basel, Basel, Switzerland; 4 Research Center for Clinical Neuroimmunology and Neuroscience Basel (RC2NB), University Hospital Basel and University of Basel, Basel, Switzerland; 5 Department of Neurology, University Hospital Basel, Basel, Switzerland; 6 Department of Medicine, University of Ottawa, Ottawa Hospital Research Institute, Ottawa, ON Canada; 7 Dipartimento Di Scienze Della Salute, Università Degli Studi Di Genova, Genoa, Italy; 8 IRCCS Ospedale Policlinico San Martino, Genoa, Italy; 9 Department of Pediatrics, Johns Hopkins University, Baltimore, MD USA

**Keywords:** POMS, CVS, PRL, 2024 McDonald criteria, Differential diagnosis

## Abstract

**Background:**

Susceptibility-based MRI biomarkers, including paramagnetic rim lesions (PRLs) and central vein sign (CVS), were incorporated into the 2024 McDonald criteria because they improve diagnostic specificity in adult-onset MS; however, their role in pediatric-onset MS (POMS) remains incompletely defined.

**Objective:**

To synthesize current evidence on the prevalence and diagnostic value of PRLs and CVS in POMS.

**Methods:**

We conducted a PRISMA-compliant systematic review and random-effects meta-analysis of studies reporting PRLs and/or CVS in POMS. Outcomes included the proportion of patients with ≥1 PRL, the mean proportion of CVS-positive lesions, and fulfillment of CVS-based thresholds.

**Results:**

Eleven studies were included. Across seven studies, the pooled proportion of POMS patients with ≥1 PRL was 71.6% (95% CI 61.1–80.2; *n*=149), with low between-study heterogeneity. Across six studies, the pooled mean proportion of CVS-positive lesions was 57.9% (95% CI 39.7–76.2; *n*=99), and 66.2% (95% CI 10.5–97.0; *n*=219) of patients fulfilled the 40%-CVS rule, with substantial variability across studies. In comparative analyses, PRLs showed high specificity for POMS, while CVS was more prevalent than in mimics; however, limited data precluded quantitative evaluation.

**Conclusion:**

PRLs are frequent in POMS. Limited comparative data suggest that PRLs may have high diagnostic specificity, whereas CVS appears more variable; however, pediatric diagnostic accuracy evidence remains scarce. Further pediatric-specific studies are needed to define the optimal diagnostic use of these biomarkers in POMS.

**Supplementary Information:**

The online version contains supplementary material available at https://doi.org/10.1007/s00415-026-14084-6.

## Introduction

Pediatric-onset multiple sclerosis (POMS) accounts for approximately 3–5% of all MS cases [1], with an annual incidence ranging from 0.07 to 2.9 per 100,000 children [2]. Compared with adult-onset MS (AOMS), POMS typically exhibits a more inflammatory phenotype, characterized by higher relapse activity and greater MRI lesion burden [3, 4]. Although recovery from acute attacks is often substantial and disability accrual tends to be slower, individuals with POMS may nonetheless reach disability milestones at a younger age due to earlier disease onset [5]. Importantly, only approximately one-third of children presenting with acquired demyelinating syndromes are ultimately diagnosed with MS [6]. Consequently, early diagnosis of POMS relies on accurately distinguishing MS from other pediatric demyelinating disorders and alternative diagnoses, including acute disseminated encephalomyelitis (ADEM), myelin oligodendrocyte glycoprotein antibody-associated disease (MOGAD), and infectious, metabolic, or genetic conditions [1]. The 2010, 2017, and 2024 versions of the international McDonald criteria for MS have formally included consideration of the diagnosis of MS in children and adolescents, and have supported the concept of MS being a single disease across pediatric and adult-onset.

Advanced MRI biomarkers, notably the central vein sign (CVS) and paramagnetic rim lesions (PRLs), have emerged as tools to improve diagnostic specificity in MS. Identified on susceptibility-based MRI, CVS reflects perivenular inflammatory demyelination, whereas PRLs represent a subset of chronically active lesions characterized by iron-laden microglia and macrophages at the lesion edge [7, 8]. In AOMS, both biomarkers improve differentiation of MS from other conditions, leading to their incorporation into the 2024 revisions of the McDonald criteria [9].

In adults, CVS is defined using the Select-6 rule, considered positive when ≥6 white matter lesions are CVS-positive, or when most lesions are CVS-positive if fewer than 10 lesions are present. In POMS, given that lesion burden is often high and that absolute lesion-count approaches have not been appropriately validated, a more conservative threshold is recommended: CVS is considered supportive of MS when ≥50% of lesions are CVS-positive [9, 10].

The McDonald panel further noted that the diagnostic value of PRLs in POMS remains uncertain due to the paucity of pediatric-specific studies.

To determine whether CVS and PRLs convey the same specificity in POMS as has been shown in AOMS, we conducted a systematic review and meta-analysis to evaluate their prevalence in POMS, critically appraise the existing literature, identify methodological limitations, and highlight priorities for future research.

## Methods

### Literature search, study selection, and data extraction

This meta-analysis was conducted in accordance with the Preferred Reporting Items for Systematic Reviews and Meta-Analyses (PRISMA) guidelines [11]. A systematic literature search was performed in PubMed in December 2025 to identify studies investigating PRLs and/or CVS in POMS. The search strategy combined terms related to MS, pediatric or early-onset populations, and susceptibility-based MRI markers of PRLs and CVS (eTable 1). Google Scholar, Embase, Web of Science and Cochrane were additionally screened as supplementary sources.

**Fig. 1:**
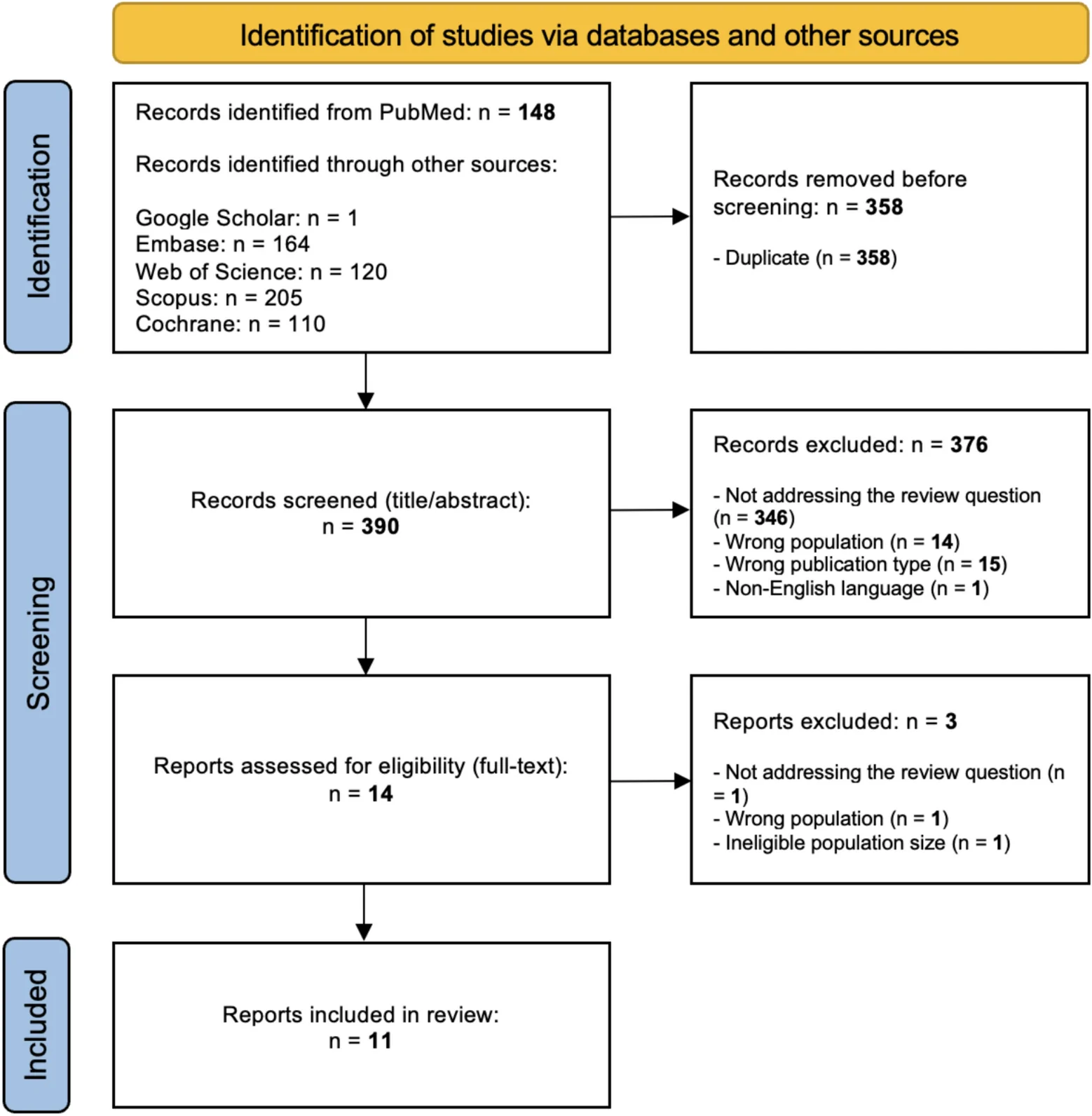
PRISMA flow diagram of study selection. Flow diagram illustrating the identification, screening, eligibility assessment, and inclusion of studies, with reasons for exclusion reported at each stage

Eligible studies were original research articles that (1) enrolled patients with POMS and (2) reported data on PRL and/or CVS prevalence. Case reports, conference abstracts, and non-English-language publications were excluded. For studies including mixed pediatric and adult cohorts, only studies reporting separate data for the pediatric population were considered eligible. The records were screened by title and abstract, followed by full-text assessment for eligibility. Study selection was performed independently by two reviewers (A.d.M. and A.C.), with discrepancies resolved by consensus. The study selection process is summarized in the PRISMA flow diagram (Fig. 1).

The same two reviewers independently extracted study-level data into a standardized database, with final entries agreed upon by consensus. When studies included additional populations in comparison with POMS, data from these populations were also extracted.

Study screening and data extraction were conducted using *Rayyan* [12]. Risk of bias and applicability concerns were assessed using the QUADAS-2 tool (eTable 2). A review protocol was not prospectively registered.

### Outcomes

For PRLs, the primary outcome was the proportion of patients with ≥1 PRL. For CVS, outcomes included (1) the mean proportion of CVS-positive lesions per patient and (2) the proportion of patients meeting the 40%-CVS-positive lesion threshold, which was selected for quantitative synthesis because it was the most frequently reported threshold across the included studies. Additional extracted data included clinical and demographic characteristics, MRI acquisition protocols, MRI analysis criteria, fulfillment of the Select-6 and 50% CVS-positive thresholds, number of raters, and measures of intra- and inter-rater agreement, when reported.

### Statistical analysis

All analyses were performed using *R* (*v. 4.3.1*). Meta-analyses were conducted using random-effects models to account for between-study heterogeneity.

The proportion of individuals exhibiting ≥1 PRL and fulfilling the 40%-CVS rule was synthesized using meta-analyses of proportions with logit transformation and inverse-variance weighting. Between-study variance was estimated using restricted maximum likelihood, and confidence intervals (CIs) were calculated using the Hartung–Knapp method, which provides more conservative inference when the number of studies is limited [13]. Sensitivity analyses included the Freeman–Tukey double arcsine transformation as an alternative to the logit transformation and leave-one-out analyses to assess the influence of individual studies.

The proportion of CVS-positive lesions per patient was pooled using a meta-analysis of means. When studies reported medians with corresponding measures of dispersion, values were approximated to means and standard deviations to allow harmonization across studies [14].

## Results

The search strategy identified 390 records. After title and abstract screening, 14 reports underwent full-text review, of which 11 were included in the quantitative synthesis (Fig. 1) [15–25]. Three studies were excluded for not addressing the review question (*n*=1), focusing on a non-POMS population (*n*=1), or reporting a single patient (*n*=1). In total, 356 patients with POMS were included across the selected studies. The main population characteristics are summarized in Table 1 with supplementary details provided in eTable 3.

**Table 1:** Study population characteristics

Study	Country	Multicentric	Assessing CVS	Assessing PRL	POMS (*n*)	Age (years)	Female (%)	Disease duration (years)	EDSS	With OCB (%)	Treated with DMTs (%)	Mimics (*n*)	Matched AOMS (*n*)
Micheletti 2022	Argentina	No	No	Yes	13	15 (SD: 2.5) {range: 9;18}	85%	NA	NA	NA	NA	16	–
Boccia 2023	Italy	No	Yes	No	10	13.56 (SD: 1.79)	60%	1.6 (SD: 1.7)	*1.0 [IQR: 1.0;2.0]	70%	100%	–	12
Harrison 2023	USA	No	Yes	No	10	11.4 (SD: 4.5)	60%	{range: 0;2.5}	*1.3	100%	NA	10	–
Margoni 2023	Italy	No	No	Yes	13	*16.2 [IQR: 12.6;16.8]	85%	*1.0 [IQR: 0.4;1.9]	*1.5 [IQR: 1.0;1.5]	100%	100%	–	–
Sacco 2023	USA	No	Yes	Yes	26	*14 [IQR: 13;17] {range: 3;17}	63%	*0.1 [IQR: 0.0;0.2] {range: 0;3.1}	*1.5 [IQR: 1.0;2.0] {range: 0;2}	85%	NA	14	–
Boccia 2024	Italy	No	No	Yes	11	16.3 (SD: 2.2)	64%	2.4 (SD: 1.5)	*1.0	91%	100%	–	–
de Deus Vieira 2024	Brazil	No	No	Yes	10	*16	80%	2.2 (SD: 1.1)	1.9 (SD: 1.3)	NA	NA	–	–
Margoni 2024	Italy	No	Yes	Yes	22	*16.4 [IQR: 13.0;17.4]	86%	*0.7 [IQR: 0.4;1.3]	*1.0 [IQR: 1.0;2.0]	100%	73%	–	–
Menascu 2024	Israel, Czech Republic	Yes	Yes	No	156	14.8	60%	NA	**2.0	NA	NA	–	–
Nistri 2025	UK	Yes	No	Yes	54	14 (SD: 2.2)	76%	*0.6 [IQR: 0.2;1.8]	NA	NA	NA	–	–
Kuchling 2025	Germany	No	Yes	No	31	13.8 (SD: 2.6)	81%	MRI at first clinical presentation	NA	97%	NA	–	–

*AOMS* adult-onset multiple sclerosis, *CVS* central vein sign, *DMT* disease modifying therapy, *EDSS* Expanded Disability Status Scale, *IQR* interquartile range, *NA* not available, *OCB* oligoclonal bands, *POMS* pediatric-onset multiple sclerosis, *PRL* paramagnetic rim lesions, *SD* standard deviation

^*^Estimates reported as medians rather than means

^**^Estimates were reported for subgroups only; no aggregate estimate for the full cohort was provided by the study

MRI timing relative to clinical presentation or disease activity varied across studies. Some studies performed MRI near the time of first clinical presentation or early in the disease course [19, 25], whereas others included patients during remission phases [18, 22, 24]. In five studies, MRI timing relative to disease activity was not specified [15–17, 21, 23].

MRI acquisition protocols varied substantially across studies, reflecting the use of both research-dedicated and routine clinical scans. Six studies were conducted on 3-T systems [16, 18, 20–23], two on 1.5-T systems [15, 25], two used a combination of 3-T and 1.5-T systems [19, 24], and one study did not report MRI acquisition parameters [17]. Susceptibility-based imaging sequences also varied substantially across studies. Detailed information on MRI protocols and sequences used across studies is provided in eTable 4. Additional details on the PRL and CVS outcomes for each included study are reported in Table 2.

**Table 2:** PRL and CVS study outcomes and assessment methods

	Study	NAIMS criteria	Outcome assessed
PRLs	Micheletti 2022	No^*^	Presence (≥ 1 PRL)
	Margoni 2023	No^**^	Presence (≥ 1 PRL)
	Sacco 2023	Yes	Presence (≥ 1 PRL)
	Boccia 2024	Yes	Presence (≥ 1 PRL)
	de Deus Vieira 2024	Yes	Presence (≥ 1 PRL)
	Margoni 2024	Yes	Presence (≥ 1 PRL)
	Nistri 2025	Yes	Presence (≥ 1 PRL)
CVS	Boccia 2023	Yes	Patient-level proportion of CVS-positive lesions; 40%-CVS rule
	Harrison 2023	Yes	Patient-level proportion of CVS-positive lesions
	Sacco 2023	Yes	Patient-level proportion of CVS-positive lesions
	Margoni 2024	Yes	Patient-level proportion of CVS-positive lesions; 40%-CVS rule; 50%-CVS rule
	Menascu 2024	Yes	40%-CVS rule
	Kuchling 2025	Yes	Patient-level proportion of CVS-positive lesions; 40%-CVS rule; Select-6 rule

^*^PRLs were defined as lesions showing a hypointense paramagnetic rim on phase images corresponding to the lesion edge on FLAIR, partially or fully encircling the lesion

^**^PRLs were defined as lesions showing a complete or incomplete rim of hypointense signal

### PRLs

Seven studies reporting PRL prevalence in POMS were included [15, 18–22, 24], comprising 149 patients, of whom 109 had ≥1 PRL. Five of seven studies applied the NAIMS criteria for PRL assessment [19, 20–22, 24].

**Fig. 2:**
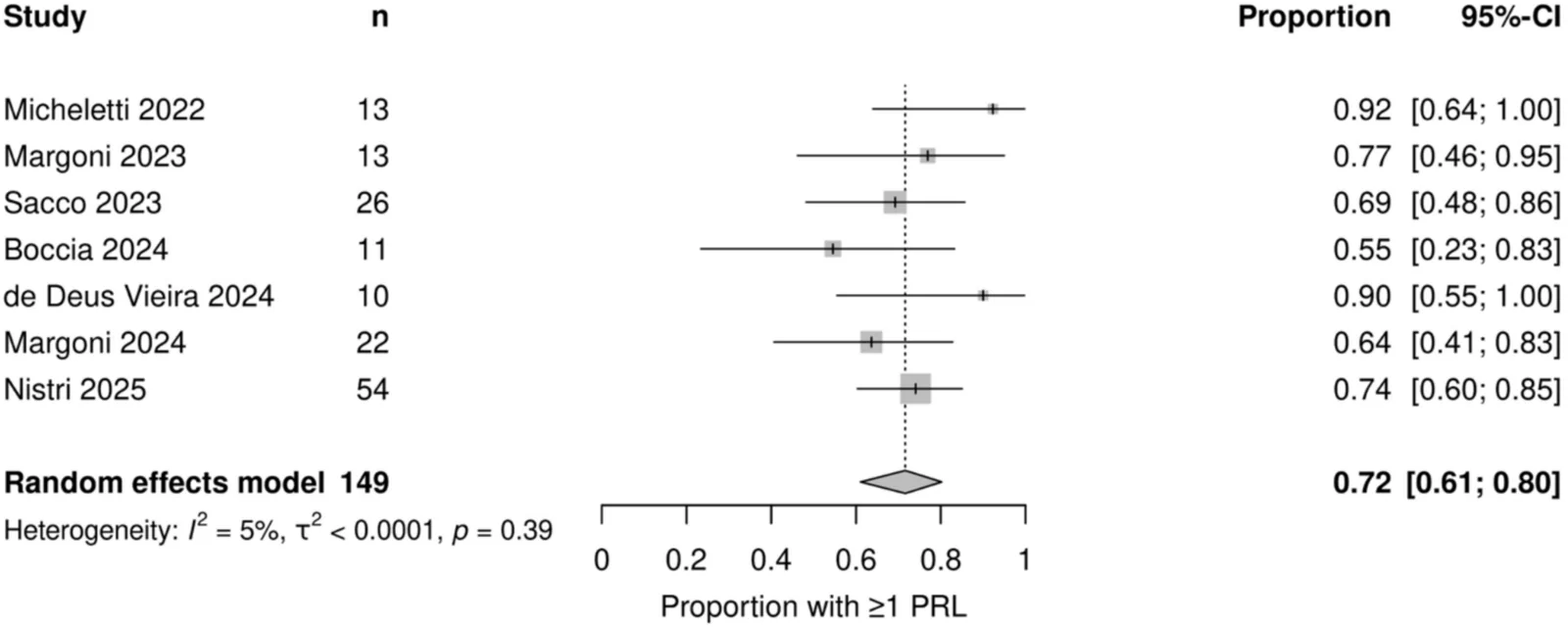
Proportion of patients presenting with PRLs in POMS. Forest plot showing study-specific proportions and the pooled random-effects estimate of the proportion of POMS patients presenting with at least one PRL. Squares represent individual study estimates with size proportional to study weight, horizontal lines indicate 95% confidence intervals, and the diamond represents the pooled estimate. Between-study heterogeneity is reported using *I*^2^ and *τ*^2^ statistics

In the random-effects meta-analysis, the pooled proportion of POMS patients with ≥1 PRL was 71.6% (95%CI 61.1–80.2) (Fig. 2). Between-study heterogeneity was low (*I*^2^=4.8%, *τ*^2^<0.0001), and the Cochran Q test was not significant (*Q*=6.30, *p*=0.39).

Sensitivity analysis using the Freeman–Tukey double arcsine transformation yielded a comparable pooled proportion of 74.1% (95% CI 63.6–83.5), with similarly low heterogeneity (*I*^2^=13.2%, *τ*^2^<0.0001; *Q*=6.91, *p*=0.33). Leave-one-out analyses showed no disproportionate influence of individual studies, with pooled estimates ranging from 70.1% to 73.2%.

### Inter- and intra-rater reliability in PRL assessment

PRL assessment was performed by two raters across all included studies. Good inter-rater reliability was reported in three studies (Cohen’s *k*=0.75 [15]; ICC=0.75 [22]; inter-rater disagreement in 4.0% of lesions [20]), and good intra-rater reliability was reported in one study (ICC=0.81) [22].

### PRL: discrimination between POMS and mimics

Two studies evaluated the performance of PRLs in discriminating POMS from pediatric MS-mimicking conditions. In the study by Sacco et al. [19], none of 14 MOGAD patients showed PRLs, whereas PRLs were detected in 18 of 26 POMS patients, yielding a sensitivity of 69% and a specificity of 100%. In the study by Micheletti et al. [15], none of 16 patients with other pediatric CNS demyelinating disorders showed PRLs, whereas 12 of 13 POMS patients had ≥1 PRL, corresponding to a sensitivity of 92% and a specificity of 100% (95% CI 93.3–100%).

### CVS

Six studies reporting CVS data in POMS were included [16, 17, 19, 22, 23, 25]. Five studies (*n*=99 patients) reported the per-patient proportion of CVS-positive lesions [16, 17, 19, 22, 25], and four (*n*=219) reported the proportion of patients meeting the 40%-CVS rule [16, 22, 23, 25]. CVS assessment followed the NAIMS criteria in all studies [16, 17, 19, 22, 23, 25].

### CVS-positive lesions per patient

**Fig. 3:**
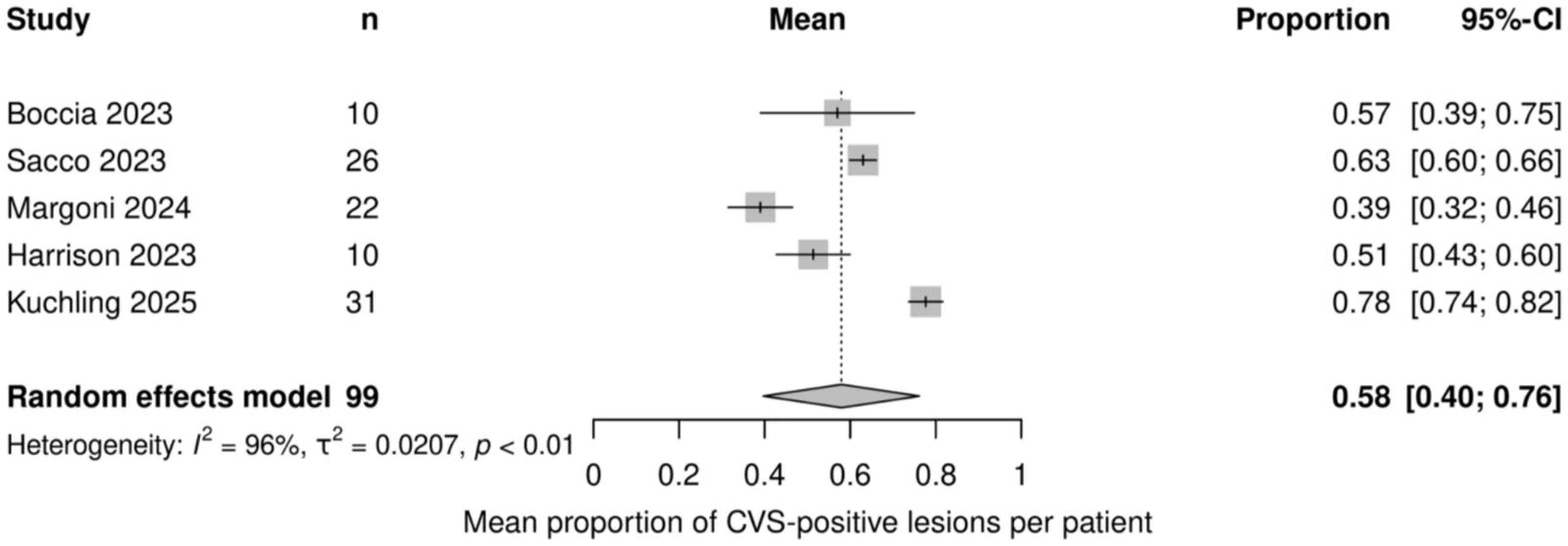
Mean proportion of CVS-positive lesions per patient in POMS. Forest plot showing the study-specific mean proportion of lesions exhibiting the CVS per patient. Squares represent individual study estimates with size proportional to study weight; horizontal lines indicate 95% confidence intervals, and the diamond represents the pooled estimate. Between-study heterogeneity is reported using *I*^2^ and *τ*^2^ statistics

In the random-effects meta-analysis, the pooled mean proportion of CVS-positive lesions was 57.9% (95% CI 39.7–76.2) (Fig. 3). Between-study heterogeneity was substantial (*I*^2^=95.9%, *τ*^2^=0.021), with significant heterogeneity on the Cochran Q test (*Q*=97.46, *p*<0.0001).

One study reported rater-specific CVS estimates with poor inter-rater agreement [17]. In the primary analysis, a single study-level estimate was derived by averaging rater-specific means and using the larger standard deviation as a conservative measure of uncertainty. Sensitivity analyses using rater-specific estimates yielded pooled mean CVS proportions of 63.8% (95% CI 42.9-84.8) for rater 1 and 52.0% (95% CI 25.1-78.8) for rater 2, both with very high heterogeneity (*I*^2^ = 97.1% and 97.7%, respectively; Q-test *p* < 0.0001). Leave-one-out analyses showed moderate variation in pooled estimates (52.5–63.2%), with persistently high heterogeneity.

### 40%-CVS rule

**Fig. 4:**
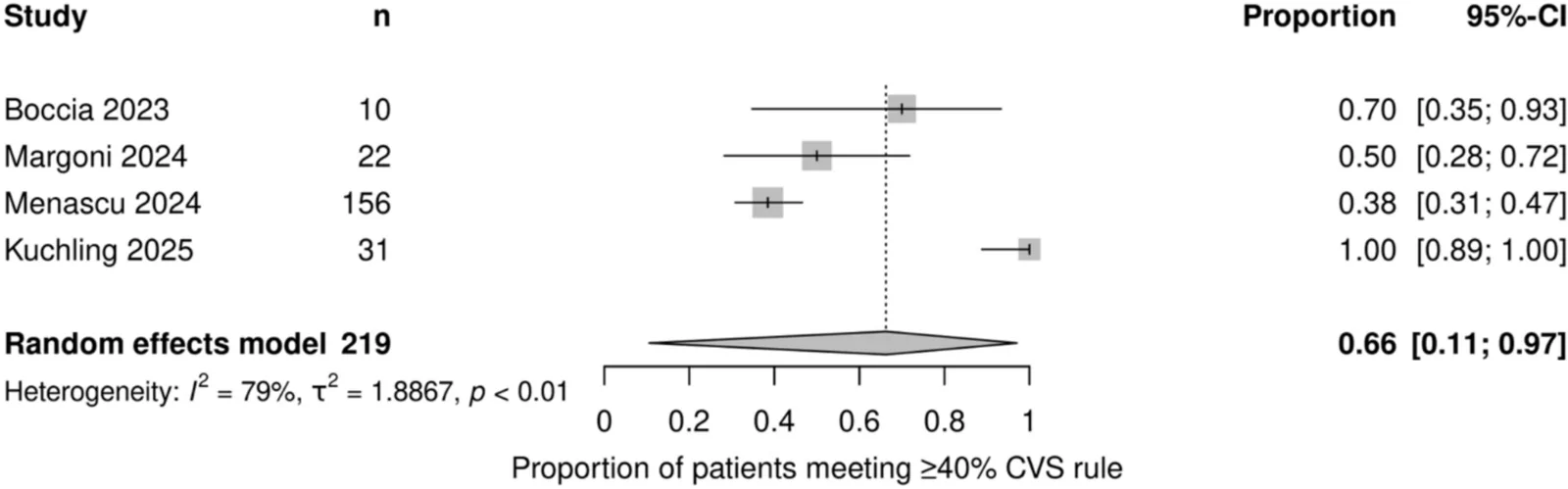
Proportion of patients meeting the 40%-CVS rule in POMS. Forest plot showing study-specific proportions of patients with POMS meeting the 40%-CVS rule. Squares represent individual study estimates with size proportional to study weight, horizontal lines indicate 95% confidence intervals, and the diamond represents the pooled estimate. Between-study heterogeneity is reported using *I*^2^ and *τ*^2^ statistics

In the random-effects meta-analysis, the pooled proportion of patients meeting the 40%-CVS rule was 66.2% (95%CI 10.5–97.0) (Fig. 4), with substantial heterogeneity (*I*^2^=78.5%, *τ*^2^=1.887; *Q*=13.96, *p*=0.003).

Sensitivity analysis using the Freeman–Tukey double arcsine transformation yielded a similar pooled proportion of 69.3% (95% CI 13.3-100.0). Leave-one-out analyses indicated instability of the pooled estimate, ranging from 47.1% (95% CI 17.5-78.9) to 78.3% (95% CI 2.3-99.8), consistent with the limited number of studies and pronounced between-study variability.

### 50%-CVS rule and Select-6 rule

Only one study evaluated the 50%-CVS rule, which was fulfilled by 5 of 22 patients with POMS (23%) [22]. Only one study assessed the Select-6 criterion, reporting fulfillment in all 31 POMS cases analyzed, including four individuals with radiologically isolated syndrome [25].

### Inter- and intra-rater reliability in CVS assessment

CVS assessment was performed by two raters in all included studies. Good to excellent inter-rater reliability was reported in three studies (ICC=0.83 [19]; ICC=0.87 [22]; percentage concordance=94.2% [23]), whereas poor inter-rater reliability was reported in one study (ICC=− 0.17 [17]). One study reported high intra-rater reliability (ICC=0.93) [22].

### CVS: discrimination between POMS and mimics

Two studies assessed CVS performance in discriminating POMS from disorders on the differential diagnosis of POMS. Harrison et al*.* evaluated 10 POMS and 10 pediatric MOGAD patients and reported higher median percentages of CVS-positive lesions in POMS than in MOGAD (rater 1: 80% vs 9.8%; rater 2: 22.7% vs. 7.5%) [17]. Similarly, Sacco et al*.* found a higher mean proportion of CVS-positive lesions in 26 POMS patients compared with 14 pediatric MOGAD patients (63% vs. 22%) [19].

One study comparing POMS with disease-duration-matched AOMS reported no difference in per-lesion CVS-prevalence (POMS: 52% vs. AOMS: 72%), but a significantly higher proportion of AOMS patients fulfilling the 40%-CVS rule (100% vs. 70%) [16].

### Combined PRL and CVS assessment

Only one study evaluated the combined prevalence of PRLs and CVS [22]. The presence of ≥1 PRL or ≥1 CVS-positive lesion was observed in 95% of POMS patients; this proportion decreased to 86% when applying a threshold of ≥1 PRL or ≥3 CVS-positive lesions, and to 77% with a threshold of ≥1 PRL or ≥6 CVS-positive lesions.

## Discussion

In this systematic review and meta-analysis, we synthesized the available evidence on the prevalence and diagnostic performance of susceptibility-based MRI biomarkers—PRLs and the CVS—in POMS. Both biomarkers were frequently detectable in POMS, although substantial variability was observed across studies for CVS-related outcomes. Although only a few studies included POMS-mimicking conditions, available evidence suggests that PRLs may have high specificity for distinguishing POMS from other disorders, whereas CVS, despite being more prevalent in POMS, appears less specific.

### PRLs in POMS

Across seven studies including 149 patients with POMS, approximately 72% harbored ≥1 PRL. Between-study heterogeneity was low, and pooled estimates were robust across sensitivity and leave-one-out analyses, indicating relatively consistent findings despite differences in MRI acquisition protocols and analytical approaches. PRL assessment showed generally good inter- and intra-rater reliability in the studies reporting these metrics, supporting the technical feasibility and reproducibility of PRL identification in pediatric populations.

In AOMS, the presence of ≥1 PRL has recently been estimated at 52% (95% CI 47–58) in a meta-analysis of 37 studies including 3180 patients, although with substantial between-study heterogeneity [26]. Notably, the pooled prevalence observed in POMS appears higher, even though some pediatric studies relied on routine clinical MRI protocols acquired at 1.5-T. While no significant effect of age on PRL prevalence was reported in the aforementioned AOMS meta-analysis [26], some studies suggest that PRL burden may decrease with increasing age and disease duration, consistent with the dynamic nature of PRLs and potential rim fading over time [27].

Beyond prevalence, two studies showed that PRLs were absent in MOGAD, NMOSD, and ADEM, resulting in 100% specificity for POMS, while sensitivity ranged from 69 to 92%. In AOMS, PRL specificity has been estimated at approximately 98% when excluding Susac’s syndrome, whereas reported sensitivity varies widely across studies (from 10 to 100%) [27], with the largest study to date reporting a prevalence of 52% [28]. The pediatric findings align with the high specificity observed in adults, although they should be interpreted cautiously given small sample sizes and limited representation of MS mimics. Interestingly, a recent study of 65 children with MOGAD identified no PRLs, providing additional support for the high specificity of PRLs in POMS [29]. Further support for a distinct biology between POMS and pediatric MOGAD is a recent study showing the absence of slowly expanding lesions (SELs), another marker of chronic active lesions, in pediatric MOGAD, while they were detected in POMS [30].

### CVS in POMS

In contrast to PRLs, CVS appeared to be less specific to POMS. The pooled mean proportion of CVS-positive lesions per POMS patient was approximately 58%, but between-study heterogeneity was very high, and pooled estimates varied considerably depending on analytical choices. Similarly, the proportion of patients fulfilling the 40%-CVS rule showed wide variability and unstable pooled estimates.

It is important to note that while CVS is detected in MOGAD and other conditions, the ability to compare the relative prevalence of CVS between different diagnoses is challenging methodologically. CVS detectability is highly sensitive to MRI field strength, spatial resolution, susceptibility contrast, and post-processing techniques. The included studies used heterogeneous MRI protocols, spanning 1.5-T and 3-T systems and a variety of susceptibility-based sequences. In addition, lesion detection criteria and analytical strategies differed substantially across studies, and inter-rater reliability was high in some studies but poor in one, [17] underscoring the operator-dependent nature of CVS assessment and the need for dedicated training in CVS interpretation.

In AOMS, the pooled proportion of CVS-positive lesions has been estimated at 73% (95% CI 67–79) in a meta-analysis of 29 studies, with reported values ranging from 58% at 1.5-T, to 74% at 3-T, and 82% at 7-T [31]. Notably, this meta-analysis also reported substantial between-study heterogeneity, mirroring the variability observed in the POMS population [31]. A study directly comparing POMS and disease-duration-matched AOMS reported a non-significantly higher proportion of CVS-positive lesions in AOMS and a significantly higher proportion of AOMS patients fulfilling the 40%-CVS rule [16]. Lower sensitivity of CVS in POMS may reflect the presence of large confluent lesions – particularly in periventricular regions – which are considered by CVS methodological criteria to be ineligible for CVS scoring.

Only one study evaluated the Select-6 rule in POMS, and only one assessed the ≥50% CVS-positive lesion threshold recommended for pediatric populations. In the latter, sensitivity decreased markedly, with only 23% of patients fulfilling the criterion, suggesting that this cutoff may be overly restrictive in some pediatric cohorts. At present, the available evidence is insufficient to determine whether specific CVS thresholds should be preferentially applied according to lesion burden or other clinical characteristics in POMS.

Only two studies formally assessed the diagnostic performance of CVS in POMS, both reporting a higher CVS prevalence in POMS than in MS-mimicking conditions. However, a recent study in a large pediatric MOGAD cohort found that 35.5% and 22.5% of patients fulfilled the 40% and 50% CVS-positive lesion thresholds, respectively [29]. These findings indicate that, although the CVS appears to be more frequent in POMS than in MS-mimicking conditions, it may also be observed in other pediatric inflammatory demyelinating disorders. Taken together, while robust evidence supports the diagnostic value of CVS in AOMS, the limited number of POMS studies warrants caution in applying CVS-based diagnostic criteria to POMS and underscores the need for pediatric-specific validation.

### Methodological limitations and gaps in the literature

Several limitations of existing literature must be acknowledged. The number of available studies is small, and most cohorts are modest in size, limiting statistical power and precision. Furthermore, multiple included studies were at high risk of bias, particularly with respect to patient selection. Potential participant overlap across studies from the same centers cannot be excluded. MRI acquisition protocols and analytical methods varied widely across studies, complicating cross-study comparisons and contributing to substantial heterogeneity, particularly for CVS. This variability also included inconsistent use of post-contrast susceptibility imaging across studies, which may influence the visibility of susceptibility-based biomarkers, particularly the CVS. Most studies were cross-sectional and retrospective, precluding assessment of lesion evolution and limiting investigation of how lesion dynamics influence the prevalence of PRLs and CVS. This is particularly relevant when assessing conditions such as MOGAD, which exhibits a high rate of lesion resolution over time. In addition, not all studies applied currently recommended NAIMS criteria for PRL assessment, as some predated their formalization. Reporting of inter- and intra-rater agreement was inconsistent, with only a subset of studies providing reliability metrics. Moreover, none of the available studies explicitly evaluated the incremental diagnostic value of PRLs or CVS beyond conventional MRI criteria.

These limitations constrained the robustness of the present meta-analysis. The small number of studies precluded meta-regression or subgroup analyses to explore sources of variability such as MRI field strength, acquisition protocols, disease duration, or analytical approaches. Consequently, pooled estimates—especially for CVS-related outcomes—should be interpreted with caution.

Further variability likely arose from differences in cohort selection, with some studies focusing on patients close to disease onset and others including more heterogeneous or longer disease durations. Finally, many studies lacked comparative pediatric MS-mimicking cohorts, limiting assessment of diagnostic specificity and comparative performance, underscoring the need for future studies incorporating well-characterized control groups within standardized diagnostic frameworks.

### Clinical implications and future directions

Taken together, our findings suggest that PRLs are a frequent and relatively consistent imaging feature in POMS. Limited comparative data suggest promising diagnostic specificity, although the available pediatric evidence remains scarce. CVS is also commonly observed in POMS, but exhibits marked variability, highlighting the need for standardized acquisition and analysis protocols and pediatric-specific validation of diagnostic thresholds.

CVS also appears to have more limited specificity, particularly when considering MOGAD. Future research should prioritize prospective multicenter studies using standardized susceptibility-based MRI protocols and standardized scoring criteria to better define the prevalence and diagnostic performance of PRLs and CVS in POMS. Studies including well-characterized pediatric MS-mimicking cohorts such as MOGAD are needed to clarify diagnostic specificity and refine pediatric-specific thresholds. In addition, longitudinal investigations will be important to characterize lesion evolution and determine how disease stage influences the detectability and clinical relevance of susceptibility-based imaging biomarkers in POMS. Overall, the findings of this meta-analysis should be interpreted with caution, as they are based on a limited number of relatively small studies. Nevertheless, they provide the first comprehensive synthesis of the available evidence on susceptibility-based MRI biomarkers in POMS.

At the same time, they emphasize the urgent need for larger, prospective, multicenter studies including relevant MS-mimicking conditions to fully establish their diagnostic value in children and to maximize their potential in clinical decision-making.

## Supplementary Information

Below is the link to the electronic supplementary material.

Supplementary file1 (DOCX 16 KB)

Supplementary file2 (DOCX 21 KB)

Supplementary file3 (DOCX 16 KB)

Supplementary file4 (DOCX 17 KB)

## Data Availability

Data in this systematic review and meta-analysis were extracted from published studies available elsewhere. Data sharing is not applicable to this article.
