# Supplementary material for: Central vein sign and paramagnetic rim lesions in pediatric-onset multiple sclerosis: a systematic review and meta-analysis
Source: J Neurol. 2026 Aug 22;273(9):540. doi: 10.1007/s00415-026-14084-6 (PMC13499817; doi:10.1007/s00415-026-14084-6)
Supplement: Supplementary file 1 [file 415_2026_14084_MOESM1_ESM.docx]

**Supplementary**

Article title: Central Vein Sign and Paramagnetic Rim Lesions in Pediatric-Onset Multiple Sclerosis: A Systematic Review and Meta-Analysis

Journal: Journal of Neurology
Corresponding author: Prof. Cristina Granziera, MD, PhD, Department of Biomedical Engineering, Translational Imaging in Neurology (ThINk) Basel, University of Basel, Hegenheimermattweg 167b, 4123 Allschwil, Switzerland. Phone: +41794877262. Fax: +41794877262.

E-mail: cristina.granziera@usb.ch

**eTable 1. Search strategy**

| Database | Search strategy |
| --- | --- |
| Pubmed | (  ("multiple sclerosis"[MeSH] OR "multiple sclerosis"[tiab] OR MS[tiab])  AND  ("child"[MeSH] OR "adolescent"[MeSH] OR child*[tiab] OR adolescen*[tiab]  OR pediatric*[tiab] OR paediatric*[tiab] OR "pediatric-onset"[tiab]  OR "paediatric-onset"[tiab] OR "early-onset"[tiab] OR juvenile[tiab]  OR POMS[tiab])  AND  (  "central vein sign"[tiab] OR "central vein"[tiab] OR CVS[tiab]  OR "paramagnetic rim"[tiab] OR "rim lesion*"[tiab] OR "iron rim"[tiab]  OR PRL[tiab] OR "paramagnetic rim lesion*"[tiab]  OR "susceptibility-weighted"[tiab] OR "susceptibility weighted"[tiab]  OR "susceptibility-based"[tiab] OR "susceptibility based"[tiab]  OR SWI[tiab] OR QSM[tiab]  )  ) |
| Embase | (('multiple sclerosis'/exp OR 'multiple sclerosis':ti,ab OR MS:ti,ab) AND ('child'/exp OR 'adolescent'/exp OR child*:ti,ab OR adolescent*:ti,ab OR pediatric*:ti,ab OR paediatric*:ti,ab OR 'pediatric-onset':ti,ab OR 'paediatric-onset':ti,ab OR 'early-onset':ti,ab OR juvenile:ti,ab OR POMS:ti,ab) AND ('central vein sign':ti,ab OR 'central vein':ti,ab OR CVS:ti,ab OR 'paramagnetic rim':ti,ab OR 'rim lesion*':ti,ab OR 'iron rim':ti,ab OR PRL:ti,ab OR 'paramagnetic rim lesion*':ti,ab OR 'susceptibility-weighted':ti,ab OR 'susceptibility weighted':ti,ab OR 'susceptibility-based':ti,ab OR 'susceptibility based':ti,ab OR SWI:ti,ab OR QSM:ti,ab)) |
| Web of science | TS=(("multiple sclerosis" OR MS)  AND (child* OR adolescent* OR pediatric* OR paediatric* OR "pediatric-onset" OR "paediatric-onset" OR "early-onset" OR juvenile OR POMS) AND ("central vein sign" OR "central vein" OR CVS OR "paramagnetic rim" OR "rim lesion*" OR "iron rim" OR PRL OR "paramagnetic rim lesion*" OR "susceptibility-weighted" OR "susceptibility weighted" OR "susceptibility-based" OR "susceptibility based" OR SWI OR QSM)) |
| Scopus | TITLE-ABS-KEY("multiple sclerosis" OR MS) AND TITLE-ABS-KEY(child* OR adolescent* OR pediatric* OR paediatric* OR "pediatric-onset" OR "paediatric-onset" OR "early-onset" OR juvenile OR POMS) AND TITLE-ABS-KEY("central vein sign" OR "central vein" OR CVS OR "paramagnetic rim" OR "rim lesion" OR "rim lesions" OR "iron rim" OR PRL OR "paramagnetic rim lesion" OR "paramagnetic rim lesions" OR "susceptibility-weighted" OR "susceptibility weighted" OR "susceptibility-based" OR "susceptibility based" OR SWI OR QSM) |
| Cochrane | ("multiple sclerosis" OR MS)  AND  (child* OR adolescent* OR pediatric* OR paediatric* OR "pediatric-onset" OR "paediatric-onset" OR "early-onset" OR juvenile OR POMS)  AND  ("central vein sign" OR "central vein" OR CVS OR "paramagnetic rim" OR "rim lesion" OR "rim lesions" OR "iron rim" OR PRL OR "paramagnetic rim lesion" OR "paramagnetic rim lesions" OR "susceptibility-weighted" OR "susceptibility weighted" OR "susceptibility-based" OR "susceptibility based" OR SWI OR QSM) |
