# Supplementary material for: Central vein sign and paramagnetic rim lesions in pediatric-onset multiple sclerosis: a systematic review and meta-analysis
Source: J Neurol. 2026 Aug 22;273(9):540. doi: 10.1007/s00415-026-14084-6 (PMC13499817; doi:10.1007/s00415-026-14084-6)
Supplement: Supplementary file 2 [file 415_2026_14084_MOESM2_ESM.docx]

**Supplementary – eTable 2 – QUADAS-2**

| **Study** | **Patient selection** | **Index**  **test** | **Reference standard** | **Flow and timing** |
| --- | --- | --- | --- | --- |
| Micheletti 2022 |  |  |  |  |
| Boccia 2023 |  |  |  |  |
| Harrison 2023 |  |  |  |  |
| Margoni 2023 |  |  |  |  |
| Sacco 2023 |  |  |  |  |
| Boccia 2024 |  |  |  |  |
| de Deus Vieira 2024 |  |  |  |  |
| Margoni 2024 |  |  |  |  |
| Menascu 2024 |  |  |  |  |
| Nistri 2025 |  |  |  |  |
| Kuchling 2025 |  |  |  |  |

+: high risk; -: low risk; ?: unclear risk
