# Supplementary material for: Central vein sign and paramagnetic rim lesions in pediatric-onset multiple sclerosis: a systematic review and meta-analysis
Source: J Neurol. 2026 Aug 22;273(9):540. doi: 10.1007/s00415-026-14084-6 (PMC13499817; doi:10.1007/s00415-026-14084-6)
Supplement: Supplementary file 3 [file 415_2026_14084_MOESM3_ESM.docx]

**Supplementary - eTable 3**

| **Study** | **Diagnostic criteria POMS** | **Diagnostic criteria comparator conditions** | **Reported age cut-off** | **Including RIS** |
| --- | --- | --- | --- | --- |
| Micheletti 2022 | 2013 IPMSSG criteria | NMOSD: Wingerchuk 2015 | 18 ys | No |
| Boccia 2023 | NR | AOMS: 2017 McDonald criteria | NR | No |
| Harrison 2023 | 2017 McDonald criteria | MOGAD: positive serum MOG cell-based assay + one ≥1 clinical CNS demyelinating event | 18 ys | No |
| Margoni 2023 | 2017 McDonald criteria |  | 18 ys | No |
| Sacco 2023 | 2017 McDonald criteria | MOGAD: positive serum MOG-live cell-based assay + CNS demyelinating event suggestive of MOGAD | 18 ys | No |
| Boccia 2024 | 2017 McDonald criteria |  | 18 ys | No |
| de Deus Veira 2024 | 2017 McDonald criteria |  | 18 ys | No |
| Margoni 2024 | 2017 McDonald criteria |  | NR | No |
| Menascu 2024 | 2017 McDonald criteria |  | 10-18 ys | No |
| Nistri 2025 | 2017 McDonald criteria |  | 18 ys | No |
| Kuchling 2025 | IPMSSG 2013 and 2017 McDonald criteria |  | 18 ys | Yes (4 patients) |

*Abbreviations: POMS, pediatric-onset multiple sclerosis; AOMS, adult-onset multiple sclerosis; NMOSD, neuromyelitis optica spectrum disorder; MOGAD, myelin oligodendrocyte glycoprotein antibody-associated disease; CNS, central nervous system; RIS, radiologically isolated syndrome; NR, not reported; IPMSSG, International Pediatric Multiple Sclerosis Study Group; ys, years.*
