# Supplementary material for: Central vein sign and paramagnetic rim lesions in pediatric-onset multiple sclerosis: a systematic review and meta-analysis
Source: J Neurol. 2026 Aug 22;273(9):540. doi: 10.1007/s00415-026-14084-6 (PMC13499817; doi:10.1007/s00415-026-14084-6)
Supplement: Supplementary file 4 [file 415_2026_14084_MOESM4_ESM.docx]

**Supplementary - eTable 4**

| **Study** | **Magnetic Field Strength (T)** | **Scanner vendor** | **Scanner model** | **Image for CVS assessment** | **Image for PRL assessment** | **Susceptibility sequence** | **Susceptibility**  **Resolution (mm)** | **Post-contrast susceptibility** | **Sequence for lesion segmentation** |
| --- | --- | --- | --- | --- | --- | --- | --- | --- | --- |
| Micheletti 2022 | 1.5 | Philips | Achieva | NA | SWI phase | SWI | 1x1x3 | Yes | 2D/3D FLAIR |
| Boccia 2023 | 3 | Siemens | Prisma | FLAIR* | NA | 3D EPI | NR | NR | 3D FLAIR |
| Harrison 2023 | NR | NA | NR | SWI | NA | SWI | Variable | NR | 3D FLAIR |
| Margoni 2023 | 3 | Philips | Achieva | NA | Phase | 3D SWI | 0.6×0.6×2.0 | NR | 3D FLAIR |
| Sacco 2023 | 3 (n=29);  1.5 (n=11) | Siemens (n=24);  GE (n=6);  Philips (n=10) | Variable | NR | NR | 3D EPI (n=14);  *swi3d1r (n=10);  SWIp (n=10);  SWAN (n=6) | Variable | Yes (n=26)  No (n=14) | FLAIR |
| Boccia 2024 | 3 | Siemens | Prisma | NA | QSM + SMWI | 3D EPI | 0.65x0.65x0.65 | NR | 3D FLAIR |
| de Deus Veira 2024 | 3 | Philips | Achieva | NA | SWI | SWI | NR | NR | 3D FLAIR |
| Margoni 2024 | 3 | Philips | Achieva | FLAIR* | Phase | 3D T2* | 0.60x0.60x2 | NR | 3D FLAIR |
| Menascu 2024 | 3 | GE | SIGNA | SWI | NA | SWI | NR | Yes | FLAIR |
| Nistri 2025 | 1.5 (n=41);  3 (n=13) | Siemens (n=45);  GE (n=6);  Philips (n=3) | NR | NA | NR | swi3d1r (n=41);  T2* (n=2);  SWAN (n=8);  SWIp (n=3) | Variable | NR | 2D/3D FLAIR |
| Kuchling 2025 | 1.5 | NR | NR | SWI | NA | SWI | NR | NR | FLAIR |

*Abbreviations: CVS, central vein sign; PRL, paramagnetic rim lesion; SWI, susceptibility-weighted imaging; SWIp, susceptibility-weighted imaging phase; SWAN, susceptibility-weighted angiography; EPI, echo-planar imaging; QSM, quantitative susceptibility mapping; SMWI, susceptibility map-weighted imaging; T2*, T2*-weighted imaging; FLAIR, fluid-attenuated inversion recovery; NA, not available; NR, not reported*
